# Morphometric Study of the Nutrient Foramina in Dry Femoral Bones at Medical Campus: An Observational Study

**DOI:** 10.31729/jnma.v63i2091.9227

**Published:** 2025-11-30

**Authors:** Anusuya Shrestha, Kumar Bhusal, Ananda Kumar Mishra, Rosha Bhandari

**Affiliations:** 1 Department of Anatomy, Maharajgunj Medical Campus, Maharajgunj

**Keywords:** *anatomy*, *femur*, *nutrient artery*, *nutrient foramen*

## Abstract

**Introduction:**

Femur is not only the longest bone, it also plays a vital role in weight transmission and locomotion. Nutrient artery, crucial in bone remodeling and fracture healing, enters the bone via nutrient foramen. This study investigates the anatomical characteristics of femoral nutrient foramina in Nepalese adults, driven by an increase in femur fractures amidst Nepal’s challenging terrain and lifestyle, including trekking, load-carrying, and agricultural activities. We aimed to study the population specific variability in the number, size, and location of nutrient foramina.

**Methods:**

The quantitative study analyzed 96 femurs (49 right, 47 left) from April to June 2025, sourced from the department of anatomy at Maharajgunj Medical College, Kathmandu. Tools like osteometric board, intravenous cannula and magnifying lens were used to measure femoral length, breadth and nutrient foramina location and size. Data were analysed using IBM SPSS Statistics version 21.0 (IBM Corp., Armonk, NY, USA)

**Results:**

Findings revealed that 56 (58.33%) of femurs had a single nutrient foramen, while 36 (37.50%) had two. The prevalence of multiple foramina on the right side was 22 (44.89%) compared to the left 14 (29.78%). Overall, 128 foramina were recorded, where 43(33.59%) sized at 22G (0.85 mm). Importantly, over 60% of the foramina were located along the linea aspera followed by medial foramina on the right side.

**Conclusions:**

The study concludes that the majority of nutrient foramina are situated along the linea aspera, with right-sided dominance in count and size, possibly due to biomechanical factors. These insights are significant for forensic/anthropological applications, including medicolegal procedures like skeletal analysis.

## INTRODUCTION

The femur is the primary weight-bearing bone of the thigh.^1,2^ It receives nutrition through four arteries: the nutrient artery, periosteal, metaphyseal and epiphyseal arteries.^1,3^ The nutrient artery penetrates mid-shaft of femur through small nutrient foramen.^4^ Nutrient arteries are important for fracture healing, bone grafting and vascularized surgeries.^5^ Variations of nutrient foramen may occur due to aging and population-specific differences or due to individual developmental variation.^1-3,6^

Nepalese people trek on steep inclines, carry heavy loads and assume prolonged squatting position imposing repetitive stress which alters the remodeling process of the femur affecting bone density and foramen position.^7,8^

The study of femoral nutrient foramen in Nepalese population is limited and none investigated the size of nutrient foramen so far. Knowledge of nutrient foramina helps safeguard the blood vessels which maintain the integrity of the bone’s internal structures.^9^ The generated data can be used for forensic investigations like age estimation.^10^

## METHODS

This was a quantitative observational study where all available bones at the study site meeting the inclusion criteria were studied using a non-probability sampling method . A total of 96 dried adult human femur bones were examined 49 from the right side and 47 from the left. These specimens were obtained from the department of anatomy at Maharajgunj Medical College, Kathmandu where the study was conducted from April to June 2025.

The bones were obtained from identified Nepalese cadavers as well as unclaimed/unidentified cadavers, which were housed in the department from past two decades. The bones of adult individuals, determined by complete epiphyseal fusion, without any visible deformities or pathology were included in the study. Ethical approval was obtained from IRC with reference number 627 (6-11E2 )/2081/82

To ensure precise measurements, various tools were utilized, including an osteometric board for measuring length of the femur, intravenous (IV) cannulae of different sizes (18G, 20G, 22G, and 24G) corresponding to outer diameters of 1.50 mm, 1.00 mm, 0.85 mm, and 0.70 mm respectively, a magnifying hand lens, and a measuring scale. Additionally, digital camera was employed to document the specimens photographically.

The femur was positioned so that the long axis of femur is aligned parallel to the osteometric board’s central axis, the femoral head facing the fixed end of the board. The sliding part of the board was brought in contact with the distal condyles of femur and the length was measured on the millimeter scale on the board. In the fixed position, femoral breadth at the level of femoral condyles (the broadest part of distal femur) was also noted. Each femur was meticulously examined using a hand lens to identify all nutrient foramina on the posterior aspect of shaft of femur. Only those foramina that penetrated the cortex obliquely and led to the nutrient canal were counted. These were identified by the presence of elevated margins around them.^11^ The location of these foramina was confirmed by gently inserting IV cannula, starting from a smaller size and increasing the size until the one with snug fit was found. At least a third of the length of cannula was inserted into the nutrient foramen leading into nutrient canal. However, the depth of the canal was not noted. This facilitated the determination of the size of each opening based on the corresponding cannula gauge. The number and precise anatomical position of each nutrient foramen was recorded by measuring its distance from the proximal end.^12^ The highest point on the head of femur was taken as the most proximal point and measured on the osteometric board directly. In cases where there were two nutrient foramina, the position of each of the foramen-proximal and distal relative to each other, were measured from the most proximal part of the head of femur. The caliber of both the proximal and distal nutrient foramina were measured using cannula. The measurements were taken by two observers at different times to ensure consistency and reliability. Foramen Index was calculated using the formula: Foramen index (FI)= (Distance from proximal end to nutrient foramen/Total length of femur) × 100

The data collected were initially organized using Microsoft Excel from Microsoft Office 365 and subsequently analyzed using SPSS software (version 21.0). Tables and graphical representations were generated using both Excel and SPSS tools to effectively present the findings.

## RESULTS

A total of 96 femur bones were examined, comprising 49 right and 47 left specimens. Mean length of femur was 426.65 mm (27.26 SD) and mean breadth at the femoral condyles was 51.86 (23.31 SD). Only 4 (4.16 %) bones lacked a nutrient foramen. Single foramen was found in 56 (58.33%) bones while the presence of two foramina was noted in 36 (37.50%) bones (Table 1).

**Table 1:** Incidence of the number of nutrient foramina in the femur (n=96).

Side	Number of Bone	Absent n(%)	One n(%)	Two n(%)
Right	49	1(2.04)	26(53.06)	22(44.89)
Left	47	3(6.38)	30(63.82)	14(29.78)

There were a total of 70 foramina on the right femurs and 58 on the left. The size analysis of individual foramina showed the caliber of 22 G (approx. 0.85 external diameter), for 29 (41.42%) of the right side foramina and for 14 (24.13%) of the left. When both sides are combined, 43 foramina (33.59%) out of a total of 128 foramina showed their caliber to be of 22 G. Moreover, the total foramina having sizes 20G (1 mm), 24 G (0.70 mm) and 18 G (1.5 mm) were 29.68%, 21.09% and 15.62 % of total foramina respectively. The individual size of the nutrient foramina of right and left sides of femur are summarized in (Table 2).

Regarding the position of the nutrient foramina, most of the foramina were found on the middle third of femur, indicated by a mean foramen index of 39.77 (12.4 SD). The foramina were found on the posterior surface and in close relation to the elevated ridge, the linea aspera as summarized in (Table 3).

**Table 2:** Incidence of the size of nutrient foramen in the femur (n=96), Total foramina (n=128).

Side	Number of Bones	Absent	Size of NF (G) n(%)			
			18G	20G	22G	24G
Right	49 (70 NF)	1	10(14.28)	21(30.00)	29(41.42)	10(14.28)
Left	47 (58 NF)	3	10(21.27)	17(36.17)	14(24.13)	17(29.78)

NF= Nutrient Foramina; G= Gauge.

**Table 3:** Situation of Nutrient Foramen on different surfaces of Femur (n=96).

Side	Number of Bones	Total no. of Foramina	On the Linea Aspera	On the Medial Side of Linea Aspera	On the Lateral Side of Linea Aspera
Right	49	70	37(52.85)	29(41.42)	4(5.71)
Left	47	58	39(67.24)	15(25.86)	4(6.89)

## DISCUSSION

The present study examined 96 human femurs (49 right, 47 left) to analyze the presence, size, and anatomical distribution of nutrient foramina. The mean length of femur in our study was 426.65 mm (27.26 SD) which is similar to South Indian (429.3 mm)^9^ and Turkish population (425.8 mm).^12^ The findings revealed that nutrient foramina were present in the vast majority of bones studied, with only 4 out of 96 bones (4.16%) lacking any foramen. This aligns closely with results reported by Mysorekar et al (3.33%)^13^ of absent nutrient foramina in femur. It has been described that in the absence of nutrient vessels, the periosteal blood vessels supply to the bone.^13^

The dominant pattern observed in both right and left femur bone was the presence of a single nutrient foramen, recorded in right (53.06%) and left (63.82%) femur, accounting for 58.33% of total. This finding is similar to Bobbili et al. (63.46%) ^9^, Padmashree et al (70%)^7^, Kizilkanat et al (88%) ^12^ and Sailakumari et al (95.6%).^5^ The presence of two foramina on the right (44.89%) compared to the left (29.78%), suggests a subtle but noteworthy side-specific variation. It suggests that although commonly, there is presence of one nutrient foramen, but when more than one foramen is found, it is more commonly seen on the right sided femur.

This asymmetry supports earlier findings by Bobbili et al., who also documented the prevalence of multiple foramina in right femurs as compared to left as 40.48% and 32.26% respectively^9^. Such variations may stem from biomechanical factors, including dominant limb use, or from intrinsic vascular development differences. This asymmetry may have implications for surgical procedures involving femoral bone interventions, warranting further investigation into its anatomical and functional significance.^14^ Our study, however, did not find more than two nutrient foramina on either right or left femur in contrast to some of the previous literature that reported the presence of multiple nutrient foramina in long bones including femur.^8,9,15,16^ This may have resulted due to the small size of the study and points towards the need of a larger study to generalize the findings.

In terms of foramen size, the current study found the 22 G caliber (approximately 0.85 mm external diameter) to be the most common, representing 33.59% of all foramina (Table 2), and frequently observed on the right femur (41.42%). This finding is in agreement with Malukar et al., who reported that mid-sized foramina were the most prevalent in Indian femurs.^15^ The second frequent caliber was 20G (29.68%), followed by the smaller (24 G- 21.09%) and the larged (18 G- 15.62%). The size variation might be influenced by hemodynamic demand, age-related remodeling, or individual genetic makeup.^6^

Anatomical mapping of the foramina revealed that 60.93% of all nutrient foramina (78 out of 128) were located on the linea aspera followed by 34.37% on the medial side of linea aspera. This resembles the findings of the other studies which state that the most common location of nutrient foramen of femur is on the linea aspera such as Mysorekar (48%)^13^, Kizilkanat (44%)^12^, Padmashree (70%)^7^, Malukar (88%)^15^and Kumar et. al. (57.32%), who emphasized the linea aspera’s role as a vascular gateway and a critical anatomical landmark for posterior femoral procedures.^9^

This study reinforces that the principal site of the nutrient foramen is on the middle third of the femur supported by a mean foramen index(FI) of 39.77. This value falls in the range of FI 33.33 to 66.66 and indicates the position in the middle third of femur (Hughe’s formula).^17^ The mean foramen index reported by other authors such as Pereira (43.7)^8^, Padmashree (43.54)^7^and Kumar (56.72)^11^ is in the similar range as ours. Moreover, some studies that have not studied foramen index have also found a major percentage of foramen on middle third of femur such as Mysorekar et al. (62%), Bobbili et al. (74.83%) and Padmashree et al. (94%). Our study invariably found the position of nutrient foramen on the posterior aspect of femur confirming that the surgical approach from anterior side of femur is safer and can prevent inadvertent vessel damage. This study also confirms the exigence of mediolateral asymmetry, particularly the increased occurrence of medial foramina in the right femurs. The data closely mirrors earlier studies, including those by Kumar et al., Mysorekar et al., and Malukar et al., adding further evidence to the importance of anatomical variations in femoral vascularization.^11,13,15^ The asymmetry in medial foramina distribution, particularly the dominance on the right side, could be attributed to repetitive mechanical stress, limb dominance, or developmental vascular patterning.

These findings have direct implications for anatomical education, and anthropological research. Furthermore, the morphometric data of femur can be used by forensic experts in stature estimation, biological profile estimation^18^ which aids in narrowing down possible matches of missing persons as well as other medicolegal procedures like identification of skeletal remains.

The study’s small sample size limits the generalizability of its conclusions and signals the need for larger, multi-center investigations to validate and expand upon these observations.

Due to inclusion of bones from unidentified/unclaimed bodies of unknown age and gender, the representativeness of the sample cannot be guaranteed and thus could show potential variability.

## CONCLUSION

The study examined the anatomical characteristics and distribution patterns of human femur nutrient foramina, emphasizing their clinical significance, and confirmed that most foramina lie along the linea aspera highlighting this ridge as the principal vascular entry point crucial for femoral perfusion. A greater frequency of both the size 22G foramina and multiple foramina was observed on the right femurs, suggesting anatomical asymmetry that could reflect limb dominance or biomechanical loading. These findings enrich anatomical database of morphometry of femur, relevant to medical education, surgery, forensics, and anthropology larger, multi-center investigations to validate and expand upon these observations.

## Data Availability

The data are available from the corresponding author upon reasonable request.
